# A Kinetic Model for Blood Biomarker Levels After Mild Traumatic Brain Injury

**DOI:** 10.3389/fneur.2021.668606

**Published:** 2021-07-06

**Authors:** Sima Azizi, Daniel B. Hier, Blaine Allen, Tayo Obafemi-Ajayi, Gayla R. Olbricht, Matthew S. Thimgan, Donald C. Wunsch

**Affiliations:** ^1^Applied Computational Intelligence Laboratory, Department of Electrical and Computer Engineering, Missouri University of Science and Technology, Rolla, MO, United States; ^2^Engineering Program, Missouri State University, Springfield, MO, United States; ^3^Department of Mathematics and Statistics, Missouri University of Science and Technology, Rolla, MO, United States; ^4^Department of Biological Sciences, Missouri University of Science and Technology, Rolla, MO, United States; ^5^ECCS Division, National Science Foundation, Alexandria, VA, United States

**Keywords:** concussion, uncertainty analysis, mathematical modeling, sensitivity analysis, blood biomarkers, kinetics, mild traumatic brain injury

## Abstract

Traumatic brain injury (TBI) imposes a significant economic and social burden. The diagnosis and prognosis of mild TBI, also called concussion, is challenging. Concussions are common among contact sport athletes. After a blow to the head, it is often difficult to determine who has had a concussion, who should be withheld from play, if a concussed athlete is ready to return to the field, and which concussed athlete will develop a post-concussion syndrome. Biomarkers can be detected in the cerebrospinal fluid and blood after traumatic brain injury and their levels may have prognostic value. Despite significant investigation, questions remain as to the trajectories of blood biomarker levels over time after mild TBI. Modeling the kinetic behavior of these biomarkers could be informative. We propose a one-compartment kinetic model for S100B, UCH-L1, NF-L, GFAP, and tau biomarker levels after mild TBI based on accepted pharmacokinetic models for oral drug absorption. We approximated model parameters using previously published studies. Since parameter estimates were approximate, we did uncertainty and sensitivity analyses. Using estimated kinetic parameters for each biomarker, we applied the model to an available post-concussion biomarker dataset of UCH-L1, GFAP, tau, and NF-L biomarkers levels. We have demonstrated the feasibility of modeling blood biomarker levels after mild TBI with a one compartment kinetic model. More work is needed to better establish model parameters and to understand the implications of the model for diagnostic use of these blood biomarkers for mild TBI.

## Introduction

Traumatic brain injury (TBI) affects 1.7 million people in the United States each year (1), placing a burden on the health care system and society. Mild traumatic brain injury (mTBI), also known as concussion, constitutes 70–90% of patients visiting an Emergency Department (ED) for TBI (2, 3). For athletes, concussive head injuries pose difficult challenges on the playing field and in the ED (4). On the playing field, it is difficult to determine which injured athlete should be withheld from play. In the ED, it is often difficult to determine which concussed patient needs a CT scan or other neuroimaging. Determining readiness to return to play after concussion is difficult. The early identification of which mTBI patients are at risk for post-concussion syndrome is imprecise. Methods are lacking to assess accumulating damage from repetitive head injuries. The prediction of chronic traumatic encephalopathy as a sequelae of mTBI is problematic. These questions have stimulated the investigation of fluid biomarkers as predictors of mTBI outcome. Fluid biomarkers were first detected in the cerebrospinal fluid (5) of patients with severe TBI. With improved assay methods these biomarkers can be detected reliably in the blood at the picogram per ml level in patients with mild TBI (6, 7).

**Figure 1:**
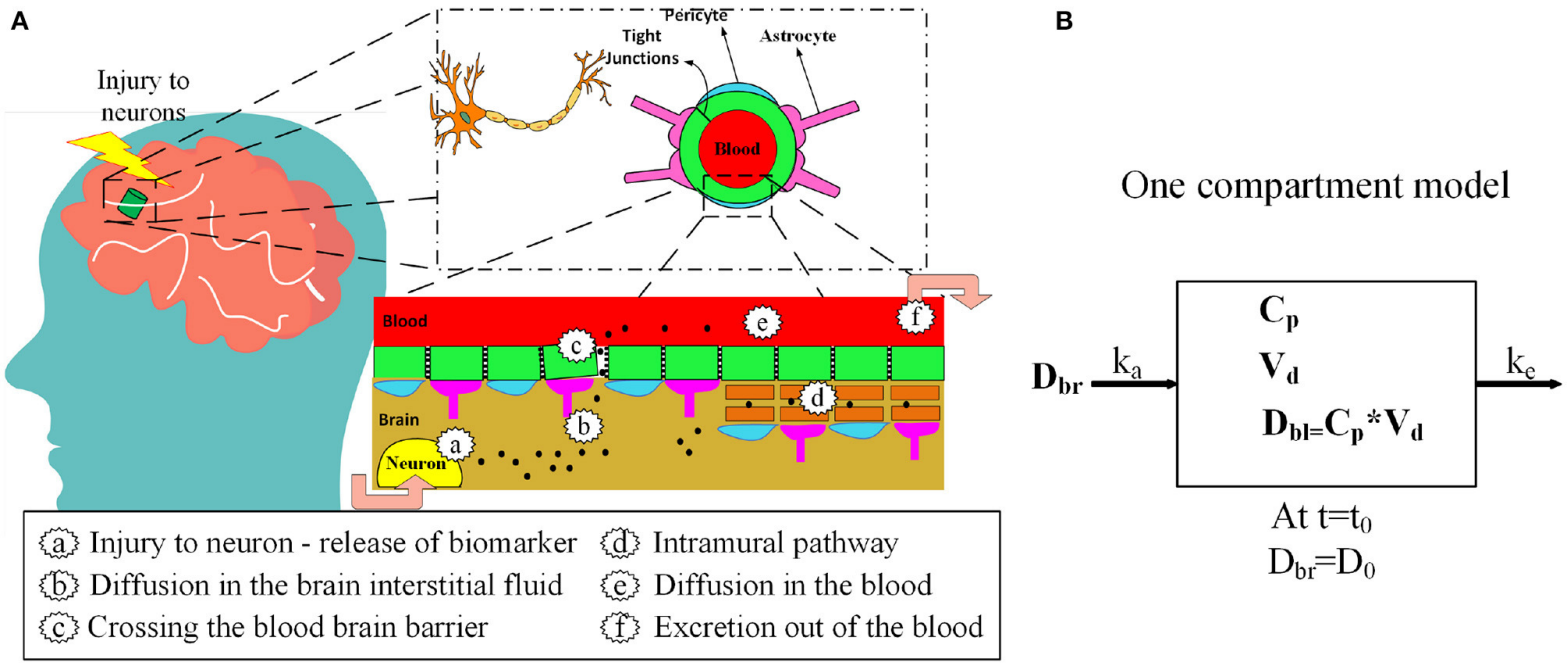
**(A)** Release of protein biomarkers (black dots) after head injury and entry into the blood via blood-brain barrier and the intramural periarterial drainage pathway. **(B)** One-compartment kinetic model for blood biomarker levels after mTBI.

Neurofilament light chain (NF-L), tau, ubiquitin C-terminal hydrolase-L1 (UCH-L1), S100B, and glial acidic fibrillary protein (GFAP) have been investigated as biomarkers for mild TBI (Table 1) (12, 13). After a concussion, neurons and astrocytes are disrupted (7) and biomarkers are released into the brain interstitial fluid (Figure 1). Although a precise relationship between impact magnitude and the amount of biomarker released has not been established, greater concussive forces are likely associated with a larger release of biomarker (22). Biomarker released into the brain interstitial fluid can reach the blood through a variety of mechanisms. Biomarker in the interstitial fluid exchanges freely with the cerebrospinal fluid where it can drain to the blood via arachnoid granulations or via lymphatic channels. Another route is direct entry into the blood via a disrupted blood-brain barrier (Figure 1). Additionally, biomarker can drain directly from the interstitial fluid to the lymphatics via arterial intramural pathways or glymphatic channels (23–32). The relative importance of each of these drainage pathways is unknown.

**Table 1:** Estimated kinetic parameters from literature review.

**Biomarker**	**Normal plasma level pg/ml**	$t_{\frac{1}{2}}$ hrs^*^	*T*_*max*_ hrs^*^	**References**
S100B	45–80	1.5^†^	2	(8–16)
UCH-L1	10–40	8	8	(8, 12, 14, 16–18)
tau	1–5	10	8	(8, 12–14, 19)
GFAP	30–70	36	24	(8, 12–14, 18–20)
NF-L	6–20	500	240	(8, 12–14, 19, 21)

* *Value for* $t_{\frac{1}{2}}$ *and T_max_ are mid-range estimates*

† *S100B is eliminated by first order kinetics. It may undergo redistribution to other compartments before renal elimination and has a shorter half-life than creatinine (15)*.

The predictive power of blood biomarkers after mTBI is under active investigation. Elevated levels of blood biomarkers can discriminate between concussed individuals and healthy controls (18, 33–35). Blood biomarkers have been investigated as predictors of neuroimaging abnormalities (abnormal CT and MRI scans) (9, 20, 35–38). Blood biomarkers have uncertain value in predicting the late effects of TBI (39–42), in detecting sub-concussive blows to the head (43–46), and in predicting readiness for return to play (47). Questions remain as to the preferred time to measure blood biomarkers, the preferred biomarker for each intended use, and cut-off values that should be used (48–53). Kinetic models of blood biomarker levels inform answers to these questions.

After a concussion, the blood level of each biomarker rises and falls over time according to its own distinct pattern (**Figure 3**). S100B rises early and falls early; NF-L rises late and falls late. *Kinetics* is the study of how the measured level of a substance changes over time. A kinetic model uses a mathematical equation to predict biomarker levels at different times. Only a few studies have created kinetic models of blood biomarker levels after mTBI (17, 54, 55). A one-compartmental kinetic model was utilized (17) to model the kinetics of ubiquitin C-terminal hydrolase-L1 (UCH-L1) levels in the cerebrospinal fluid and blood after severe TBI. Ercole et al. (54) modeled S100B levels after TBI with a hierarchical, Bayesian gamma variate kinetic equation. Dadas et al. (55) used MATLAB® to build a multi-compartment pharmacokinetic model to predict blood biomarkers levels after TBI and to model disruption of the blood barrier.

**Figure 2:**
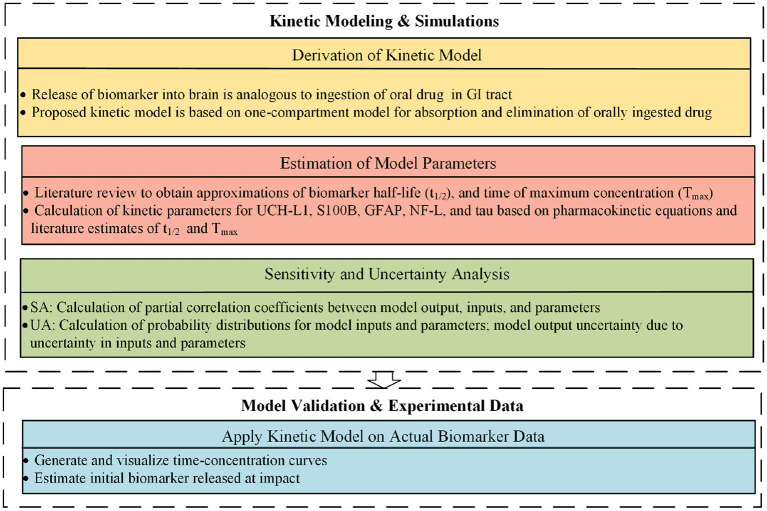
Proposed approach.

We propose a one-compartment kinetic model (Figure 2) to predict blood levels of the biomarkers S100B, UCH-L1, NF-L, GFAP, and tau after mTBI. The kinetic model provides estimates of blood biomarker levels at different times. If biomarker levels are known at specific times, an estimate of initial release of biomarker at time of impact can be provided. Since precise model parameters are not available, we approximated kinetic parameters based on a review of published studies and used sensitivity analysis and uncertainty analysis to assess the implications of estimation errors on model accuracy. We applied the model to an available dataset of post-concussion biomarker levels (56, 57).

## Methods

### Kinetic Model

The proposed kinetic model is based on a standard one-compartment model for the oral absorption of a drug from the GI tract into the blood (58). In a one-compartment model, ingested drug is absorbed from the GI tract into the blood by first order kinetics. First order kinetics assumes that the amount of drug entering the blood per unit time is the amount of drug in the GI tract multiplied by a rate constant *k*_*a*_. Once in the blood, the drug is eliminated by first order kinetics so that the amount of drug eliminated per unit time is the amount of drug in the blood multiplied by the elimination rate constant *k*_*e*_. Not all of the drug in the GI tract enters the blood. The fraction entering the blood is a unit-less ratio called bioavailability or *F*. The model is considered *one-compartment* because drug that enters the blood compartment stays there until elimination and is not redistributed to other compartments such as the fat or interstitial fluid. After ingestion of a drug there are two primary kinetic phases, an absorption phase when absorption outpaces elimination and an elimination phase when elimination outpaces absorption. The kinetic parameter *T*_*max*_ describes the time when drug levels are at a peak. *T*_*max*_ marks the end of the absorption phase and the beginning of the elimination phase. The maximum drug level at *T*_*max*_ is called *C*_*max*_. Another important kinetic parameter is the *half-life* or $t_{\frac{1}{2}}$ which is the time for blood levels to fall by 50% during the elimination phase after absorption has ceased. In our model, the release of a biomarker into the brain compartment is analogous to the ingestion of a drug into the GI tract. For both models, F is the bioavailability (fraction of drug or biomarker entering the blood), *k*_*a*_ is the first order absorption rate constant for entry into the blood, and *k*_*e*_ is the first order rate constant for elimination from the blood. Similarly, for both the drug and biomarker model, *C*_*p*_ is the plasma concentration and *V*_*d*_ is the volume of distribution (59). Model inputs include:

- *D*_0_, the amount of biomarker released into the brain at time of impact *t*_0_. (The model assumes release is complete and simultaneous at *t*_0_ with no timed or delayed release).
- *V*_*d*_, the volume of distribution (we have approximated *V*_*d*_ as total blood volume).

Model parameters that depend upon the biomarker modeled include:

- *F*, the fraction of biomarker entering the blood from the brain.
- *k*_*a*_, the first order rate constant for absorption of biomarker into the blood from the brain compartment.
- *k*_*e*_, the first order rate constant for the elimination of biomarker from the blood.

Model variables that vary as a function of time include:

- *D*_*bl*_, the amount of biomarker in the blood at time *t*.
- *D*_*br*_, the amount of biomarker in the brain at time *t*.

The model output is the blood biomarker level *C*_*p*_. In a one-compartment model, the net change in the amount of biomarker in the blood at time *t* is equal to inflow minus outflow (Equation 1). The difference between inflow and outflow is an ordinary differential equation (Equation 2).

(1) $$\begin{array}{l} net=inflow-outflow \end{array}$$

(2) $$\begin{array}{l} inflow=F*k_{a}*D_{br}=F*k_{a}*D_{0}*e^{-k_{a}t}\\ outflow=k_{e}*D_{bl}\\ net=\frac{dD_{bl}}{dt}=F*k_{a}*D_{0}*e^{-k_{a}t}-k_{e}*D_{bl} \end{array}$$

Solving Equation (2) for *D*_*bl*_, the blood concentration *C*_*p*_ of a biomarker at time *t* is modeled as a bi-exponential equation (Equation 3):

(3) $$\begin{array}{l} C_{p}=\frac{D_{bl}}{V_{d}}\\ C_{p}=\frac{F*D_{0}*k_{a}}{V_{d}*\left(k_{a}-k_{e}\right)}*\left(e^{-k_{e}t}-e^{-k_{a}t}\right) \end{array}$$

### Estimation of Kinetic Parameters

We used published estimates of *T*_*max*_ (time of maximum concentration) and $t_{\frac{1}{2}}$ (half-life; Table 1) to estimate *k*_*a*_ and *k*_*e*_ for each biomarker (Equations 4 and 5) based on the assumption of first order kinetics (59). Values in Table 1 are mid range estimates from reported values. If half-life or *T*_*max*_ was not specifically mentioned in a research report and a usable time-concentration curve was available we used the method of Thelin et al. (8) to estimate half-life as the time required for reported levels to drop by 50% and *T*_*max*_ as the time at which biomarker concentration was at its peak.

(4) $$\begin{array}{l} t_{\frac{1}{2}}=\frac{0.693}{k_{e}} \end{array}$$

(5) $$\begin{array}{l} T_{max}=\frac{\ln\;\left(\frac{k_{a}}{k_{e}}\right)}{k_{a}-k_{e}} \end{array}$$

Values for *F* (the fraction of biomarker released into the brain that reaches the blood) are likely to vary by biomarker and are not available in the literature. Based on the impermeability of the brain barrier to proteins, it was previously thought that *F* was low (perhaps 0.01 to 0.05) for most biomarkers (55). We used a higher estimate of 0.8 based on the high recovery of biomarker in the blood and lymph after intra-brain and intra-ventricular injection of biomarker in animals experiments (28, 60–62).

**Figure 3:**
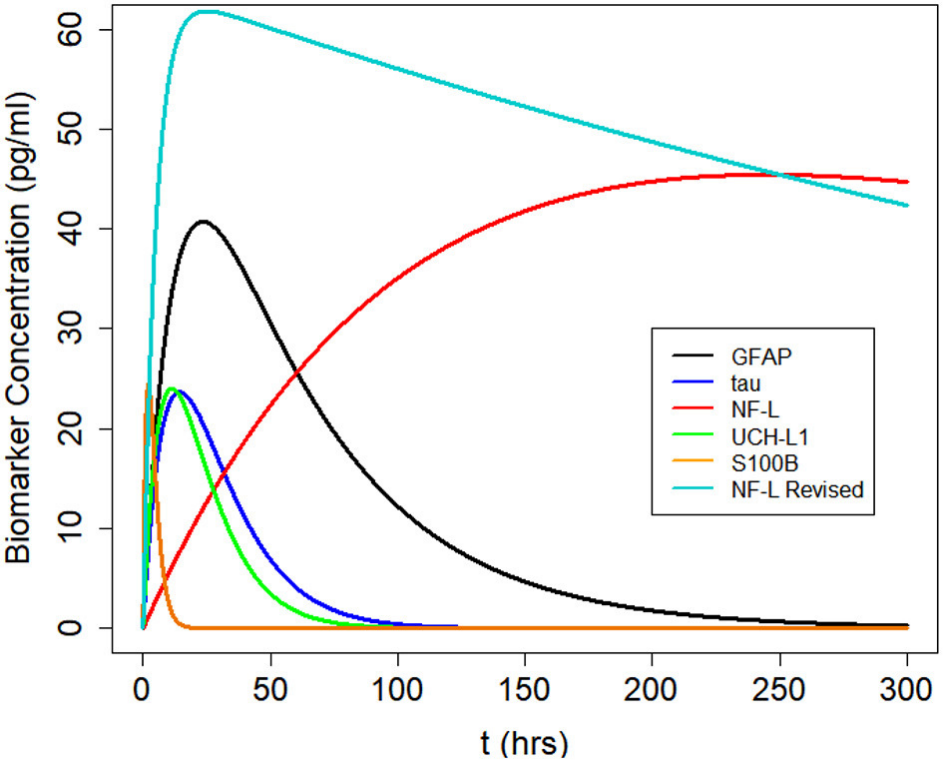
Kinetic profiles for five blood biomarkers with parameter values for *k*_*a*_ and *k*_*e*_ from Table 2, a nominal total blood volume of *V*_*d*_ = 5, 000 ml, and a stipulated biomarker release of biomarker *D*_0_ = 400, 000 pg. Kinetic profiles based on Equation (3). The revised NF-L curve reflects a higher *k*_*a*_ that provides a better fit to the NF-L levels in the NCAA-CARE dataset.

We used Equation (3) to create five models, one for each of the biomarkers, to estimate blood levels at time *t* after concussion (Figure 3 and Table 2). Model parameters *k*_*a*_, *k*_*e*_, and *F* are biomarker-dependent and we used estimates from Table 1. *V*_*d*_ and *D*_0_ are concussion-dependent and vary by the individual sustaining a concussion. As a simplification, we used a nominal value of 5,000 ml for *V*_*d*_, although total blood volume differs by individual (63, 64). *D*_0_ is the unknown amount of biomarker released into the brain at time of concussion and varies according to the severity of the concussion. For Figure 3 we stipulated a nominal release of 400,000 pg of biomarker at concussion.

**Table 2:** Kinetic models with calculated parameters.

**Biomarker**	**Parameter**			**Input**	
**model**	*k*_e_^*^	*k*_a_^*^	***F*^†^**	**Nominal *D*_0_**	**Nominal *V*_*d*_**
S100B	0.462	0.5	0.8	400,000	5,000
UCH-L1	0.0866	0.09	0.8	400,000	5,000
tau	0.0693	0.07	0.8	400,000	5,000
GFAP	0.0193	0.08	0.8	400,000	5,000
NF-L	0.0014	0.009	0.8	400,000	5,000
NF-L Revised	0.0014	0.2	0.8	400,000	5,000

* *Units for rate constants are hr^−1^*.

† *F is bioavailability and is a unit-less ratio. Estimates for F are not available in literature*.

*Value was stipulated to be 0.8. Actual values are likely to differ*.

### Sensitivity and Uncertainty Analysis

**Figure 4:**
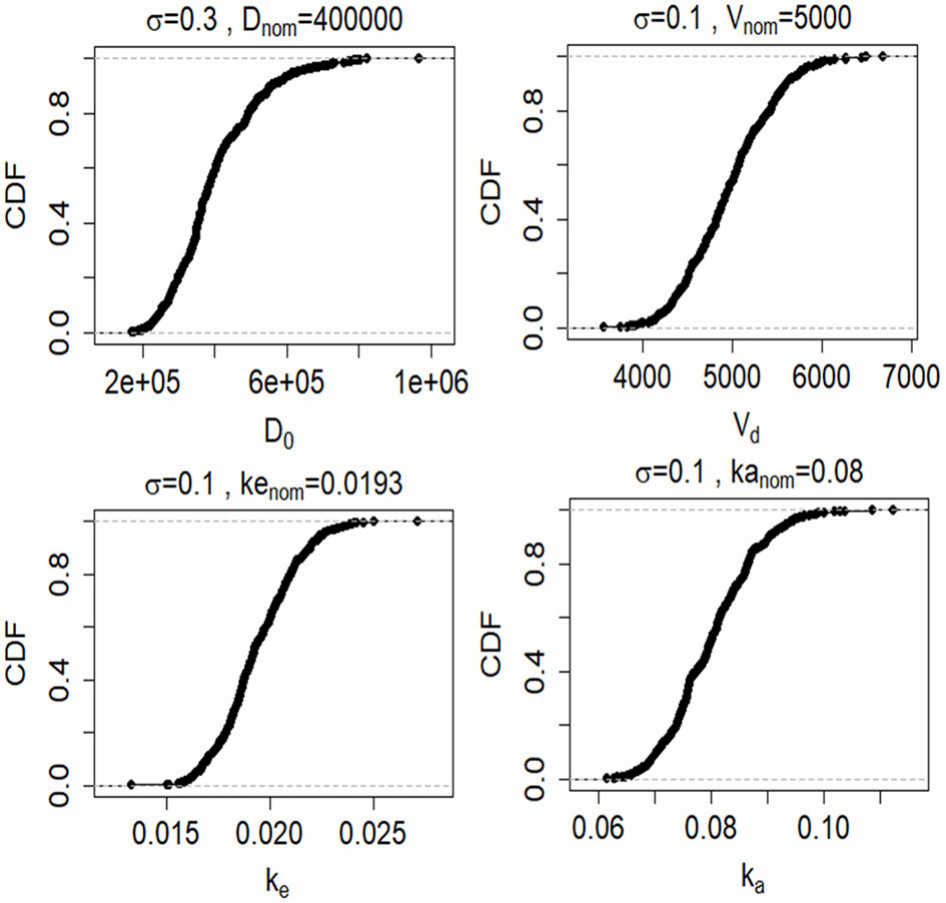
Cumulative distribution functions (CDFs) of 1,000 samples randomly drawn from lognormal probability distributions (described by Equation 7) for the four model inputs and parameters (*D*_0_, *V*_*d*_, *k*_*e*_, and *k*_*e*_). CFs are shown for the GFAP kinetic model.

**Figure 5:**
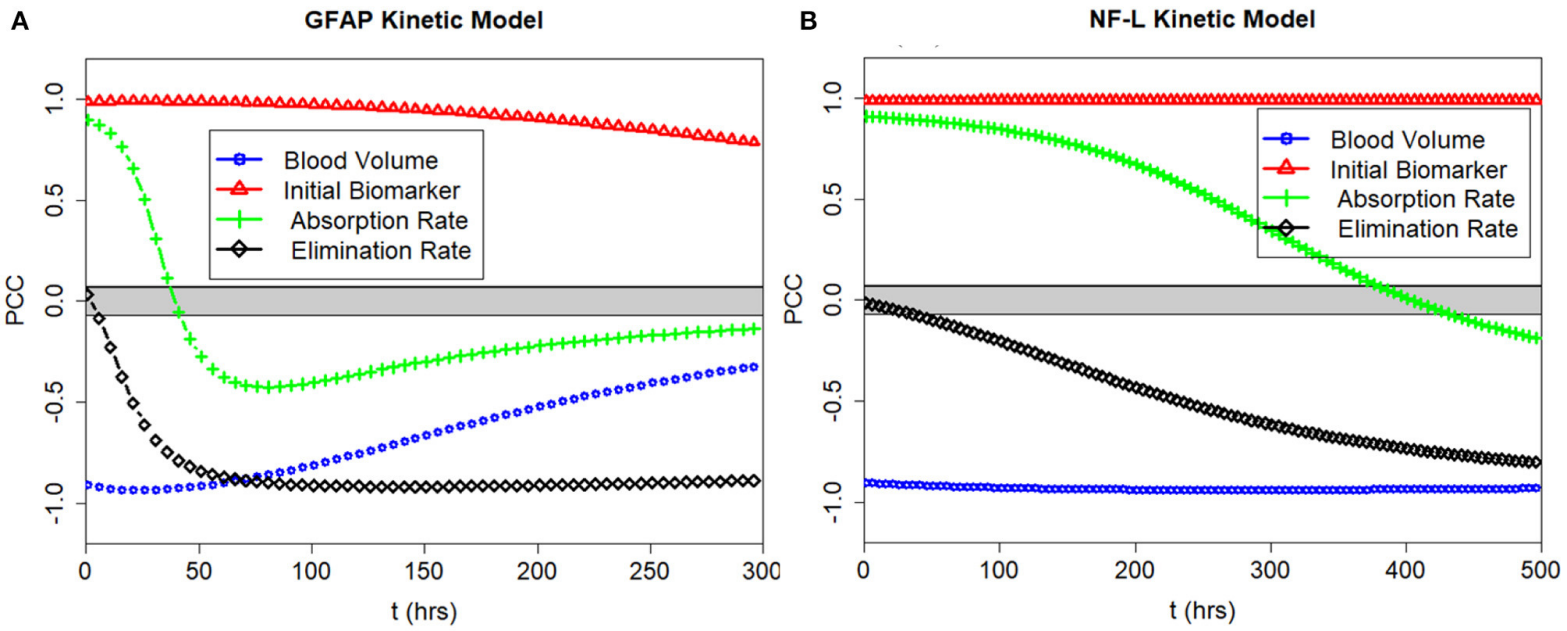
PCC of the model output with the input parameters *k*_*e*_ and *k*_*a*_ and the model inputs *V*_*d*_ and *D*_0_ plotted over time. **(A)** Correlations for GFAP model. **(B)** Correlations for NF-L model. Partial correlations near +1.0 and −1.0 show strong correlations between input and output. Partial correlations near 0.0 show weak correlations. Note that *D*_0_ correlates positively with *C*_*p*_ at all times, and that *V*_*d*_ correlates negatively with *C*_*p*_ at all times. Partial correlations with *k*_*e*_ and *k*_*a*_ depend on time of measurement.

To assess the validity of our model, we performed both a sensitivity analysis and uncertainty analysis to explore how uncertainty about model inputs (*D*_0_, *V*_*d*_, and *t*) and model parameters (*k*_*e*_, and *k*_*a*_) influences the output (*C*_*p*_). For the sensitivity analysis (65), we calculated the partial correlation coefficient between model inputs (*V*_*d*_, *D*_0_, *k*_*a*_, and *k*_*e*_) and model output (*C*_*p*_) as a function of time (**Figure 5**). We used the partial correlation coefficient to assess the linear relation between a single model input and the model output after adjusting for the effects of the other model inputs (66). We tested the significance of the partial correlation coefficient (γ) using a t-distribution (Equation 6).

(6) $$\begin{array}{l} T=\gamma \sqrt{\frac{N-2-p}{1-\gamma^{2}}}\sim t_{N-2-p}, \end{array}$$

where *t*_*N*−2−*p*_ is the t distribution with the *N* − 2 − *p* degrees of freedom, N is the sample size and *p* is the number of varied input variables minus one (*k* − 1). The null hypothesis is that partial correlation coefficient is equal to zero and is rejected if the absolute value of the test statistic is higher than the 1 − α/2 percentile of a t-distribution with N-2-p degrees of freedom (where α is the significance level). We created a probability density function for each of the model inputs based on nominal values from Table 1. Since our model inputs are constrained to be positive, we chose a lognormal distribution for the probability density function:

(7) $$p_{i}\sim \text{lognormal}\;(\log(p_{nom,i})-\frac{\sigma_{i}^{2}}{2},\sigma_{i}^{2}),$$

where *p*_*nom,i*_ is the nominal value of parameter *i* obtained from Table 1 and σ_*i*_ is its associated variance. We created lognormal probability density functions for each model input for each of the five biomarkers (Figure 4). We assessed how uncertainty in the model inputs or parameters propagated to uncertainty in model output using Monte Carlo simulations. For each Monte Carlo simulation we created a matrix of 1,000 rows × *k* columns, where *k* was the number of inputs or parameters in the model that we varied simultaneously and ranged between 1 and 5. Each row was created by randomly sampling the values from the probability distribution function for parameter of interest (Figure 4). Each column reflected a different parameter or input. *C*_*p*_ was calculated 1,000 times (Equation 3). All other model inputs were set to their nominal values.

### Estimation of Biomarker Released

The biomarker data utilized for model validation is drawn from the NCAA Concussion Assessment, Research, and Education (CARE) study (67), which is available via the Federal Interagency Traumatic Brain Injury Research (FITBIR) (68) data repository to approved investigators [the data set was downloaded from FITBIR (68) on August 22, 2019]. The study was established by the National Collegiate Athletic Association and the US Department of Defense. For a subset of concussed subjects, blood biomarker data (NF-L, tau, UCH-L1, and GFAP) were obtained at 6 h and at 24–48 h after injury, when asymptomatic, and at 7 days after return to play. Based on nominal values for our kinetic model parameters *F*, *k*_*a*_, *k*_*e*_, and *V*_*d*_, we derived values of *C*_*p*_ from the available biomarker data and Equation (3) to estimate the amount of biomarker released (*D*_0_) for each concussed subject in the data set. Note that the data is only utilized for model validation since the primary focus in this work is model design and simulation.

## Results

### Sensitivity Analysis

We calculated the partial correlation coefficient between the model output *C*_*p*_ and the model parameters *k*_*e*_, *k*_*a*_ and the model inputs *V*_*d*_ and *D*_0_ over time. The partial correlation coefficients for GFAP and NF-L are shown in Figure 5. The partial correlation coefficient varies between −1 (strong negative correlation) and +1 (strong positive correlation). The gray band is the area where coefficients are not significantly different from zero (obtained using the test statistic in Equation 6). For GFAP (Figure 5A), at early time intervals (0–6 h), *C*_*p*_ is most correlated with volume of distribution (*V*_*d*_), initial biomarker release (*D*_0_) and absorption rate (*k*_*a*_). At later time intervals (30–50 h) GFAP level correlates most with volume of distribution, initial biomarker release, and elimination rate (*k*_*e*_). Absorption rate (*k*_*a*_) is more determinate of biomarker level early on whereas elimination rate (*k*_*e*_) is more determinate in later time intervals between 30 and 50 h. As expected, the correlation between GFAP level and absorption rate is positive, the correlation between GFAP level and elimination rate is negative. The same pattern is true for the NF-L kinetic model (Figure 5B), except that the timing differs. Due to lower absorption rate (*k*_*a*_) and lower elimination rate (*k*_*e*_) the partial correlation curves are shifted to the right suggesting that absorption rate is an important determinate of blood biomarker levels for longer after a concussion and that elimination rate becomes an important determinate of blood biomarker levels later after a concussion (compared to GFAP). The partial correlation coefficients are also calculated for two other biomarkers of tau and UCH-L1. Due to the similarity between the pattern of GFAP with tau and UCH-L1, their partial correlation coefficient curves over time are not included in Figure 5.

### Model Uncertainty Due to Joint Uncertainty in *k*_*e*_, *k*_*a*_, *V*_*d*_, and *D*_0_

**Figure 6:**
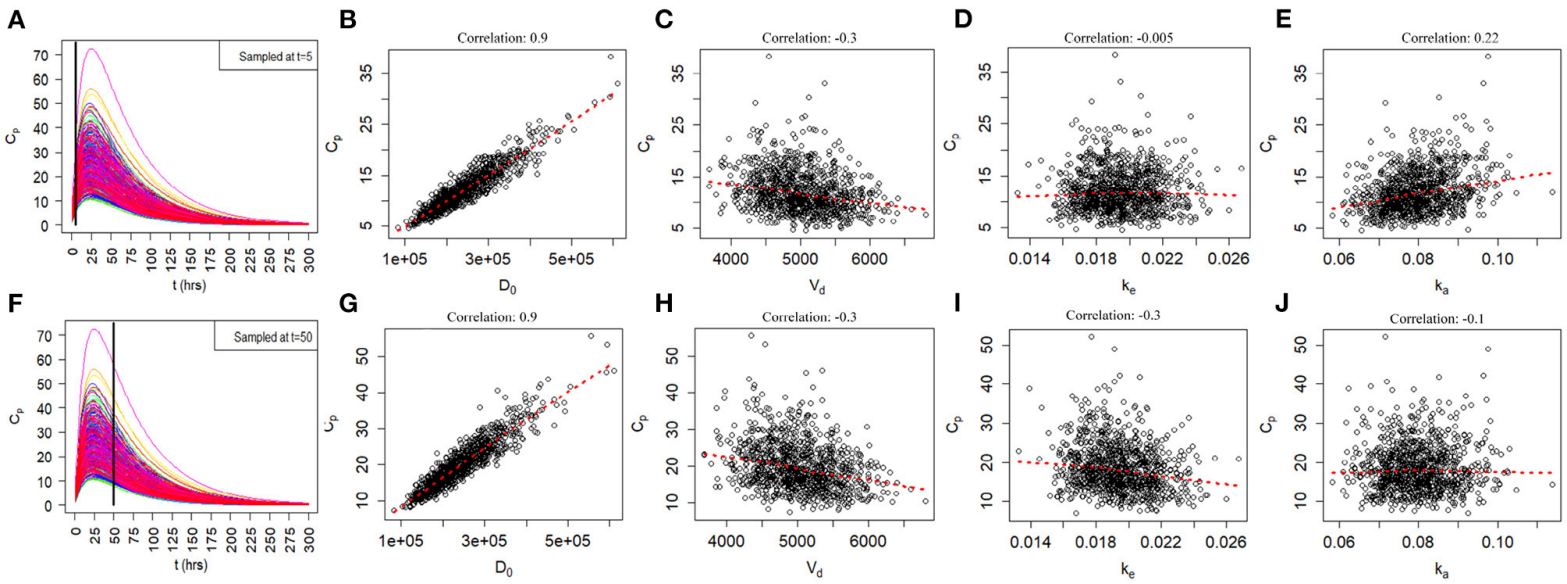
Monte Carlo simulations to investigate relationship of joint uncertainty in *D*_0_, *V*_*d*_, *k*_*e*_, and *k*_*a*_ on model output for the GFAP model. One thousand runs are shown. **(A–E)** Scatter plots of model output by input and parameter at 5 h. **(F–J)** Scatter plots of model output by input and parameter at 50 h. Model output (Cp) correlates positively with biomarker release *D*_0_ and negatively with *V*_*d*_. Uncertainty in estimates of *k*_*a*_ and *k*_*e*_ have a smaller influences on model output. The positive correlation of *k*_*a*_ with *C*_*p*_ is most apparent at 5 h and the negative correlation of *k*_*e*_ with *C*_*p*_ is most apparent at 50 h.

Since the exact values for some of the model inputs and parameters are uncertain, we created a probability distribution based on the nominal value of the model inputs and parameters. We described the probability distribution of volume of distribution, absorption rate, and elimination rate as a lognormal distribution (Equation 7). The nominal value for model inputs and parameters were set to the values in Table 2. The cumulative distribution functions of the model inputs are shown in Figure 4. We varied the initial biomarker release (*D*_0_) over a larger range (σ = 0.3), while other inputs such as *V*_*d*_, *k*_*a*_, and *k*_*e*_ varied with a smaller standard deviation of (σ = 0.1). The cumulative distribution functions in Figure 4 are shown for the GFAP kinetic model. Similar cumulative distribution functions were calculated for each biomarker. To examine model uncertainty due to joint uncertainty in *k*_*e*_, *k*_*a*_, *V*_*d*_, and *D*_0_, we performed a Monte Carlo simulations with 1, 000 random samples taken from the cumulative function distributions of the two model inputs and two model parameters (Figure 4). Results for the GFAP kinetic model are shown in Figures 6A,F. Scatter plots in Figures 6B–E,G–J demonstrate the relation between the output (*C*_*p*_) and the model inputs at two different times (Figures 6A,F). The first row corresponds to *t* = 5 h and the second row corresponds to *t* = 50 h. The red dashed lines are the smoothed LOESS fits (69) identify linear, nonlinear and correlations between the model outputs and inputs. For GFAP, a positive linear correlation is observed between the model output and the initial blood biomarker released at time-points *t* = 5 h and *t* = 50 h. A negative correlation is found between model output and total blood volume. The correlation between the model output and absorption and elimination constants are smaller when compared to the model inputs of biomarker released and volume of distribution, suggesting that these model parameters have less influence on predicted biomarker levels than the model inputs *D*_0_ and *V*_*d*_. Further, the relation between these two model parameters and the output varies with time. For instance, there is little relation between model output and elimination rate at *t* = 5 h (Figure 6D). However, a negative correlation is observed at *t* = 50 h. Similarly, there is little relation between absorption rate and the model output at *t* = 50 h, and a positive correlation is observed at *t* = 5 h.

### Model Uncertainty Due to Uncertainty About *k*_*a*_ and *k*_*e*_

**Figure 7:**
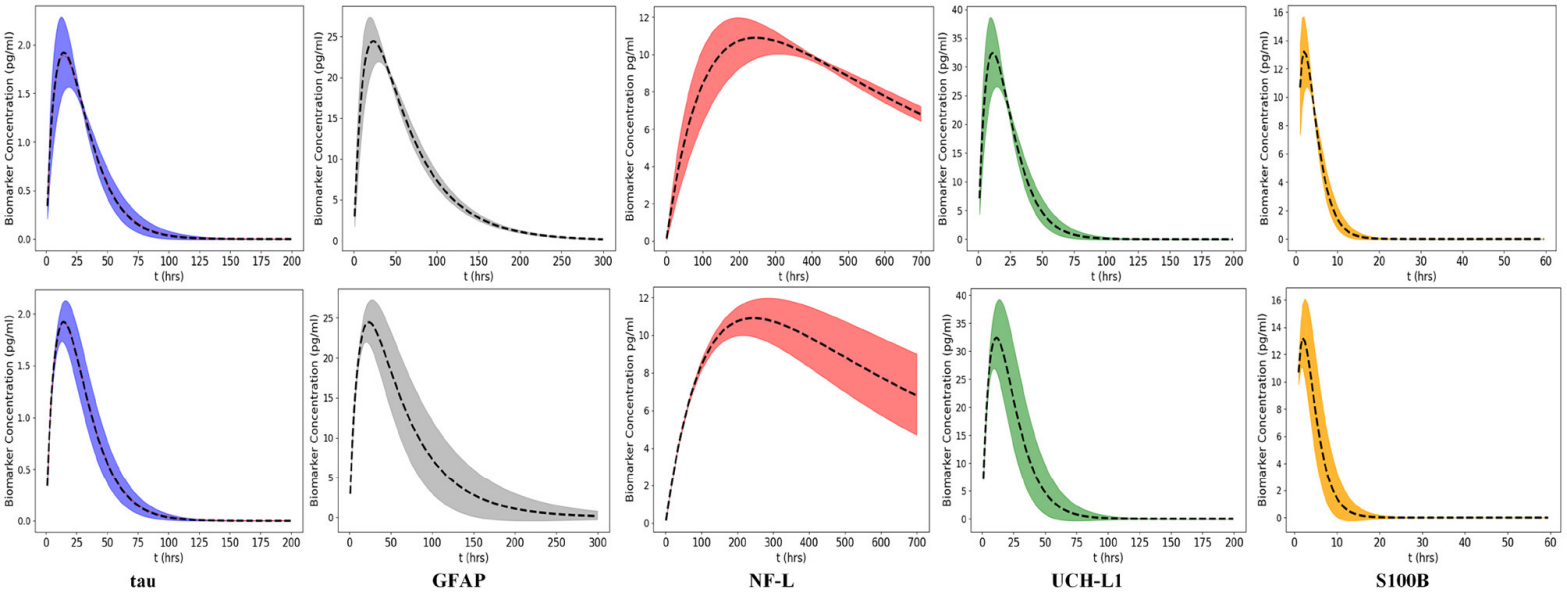
Uncertainty propagated in the model output caused by variation of two input parameters *k*_*a*_ and *k*_*e*_. The uncertainty bands are the 2δ uncertainty of the model output (δ is the standard deviation of the model output). Variations in *k*_*a*_ and *k*_*e*_ are derived from the lognormal probability distributions (defined by Equation 7), the standard deviation is 0.2 **(Top)** Model output uncertainty due to *k*_*a*_. **(Bottom)** Model output uncertainty due to *k*_*e*_. Peak of each curve corresponds to *C*_*max*_ at *T*_*max*_. Curve up to *T*_*max*_ is absorption phase and curve after *T*_*max*_ is elimination phase. Uncertainty in *k*_*a*_ dominates absorption phase whereas uncertainty in *k*_*e*_ dominates the elimination phase for all biomarkers. Time intervals of greatest uncertainty depend on the specific values of *k*_*e*_ and *k*_*a*_. Note that the x-axis has been expanded or compressed depending on the half-life of the biomarker. Confidence limits provide insight as to when the uncertainty about *k*_*a*_ or *k*_*e*_ causes greatest uncertainty about biomarker level *C*_*p*_.

We used a Monte Carlo simulation to explore the propagation of uncertainty about *k*_*a*_ and *k*_*e*_ to the model output. We varied *k*_*a*_ and *k*_*e*_ over their lognormal distribution values (standard deviation set to σ = 0.2 while setting the other models inputs to their nominal values (Table 2). At each time point, model output was evaluated using 1, 000 random values for *k*_*a*_ or *k*_*e*_. The confidence bands are defined as the ±2δ uncertainty of the model output (δ is the standard deviation of the model output, Figure 7). The upper row shows the confidence bands for *k*_*a*_ and the lower row shows the confidence bands for *k*_*e*_. The confidence bands of the NF-L biomarker model are extended for a longer period of the time (*t* = 700 h) because elimination of this biomarker is slower. For all biomarkers, model output uncertainty due to uncertainty in *k*_*a*_ occurs during the absorption phase (rising levels) and output uncertainty for *k*_*e*_ occurs during the elimination phase (falling levels; Figure 7).

### Model Uncertainty Due to Uncertainty About *t*

**Figure 8:**
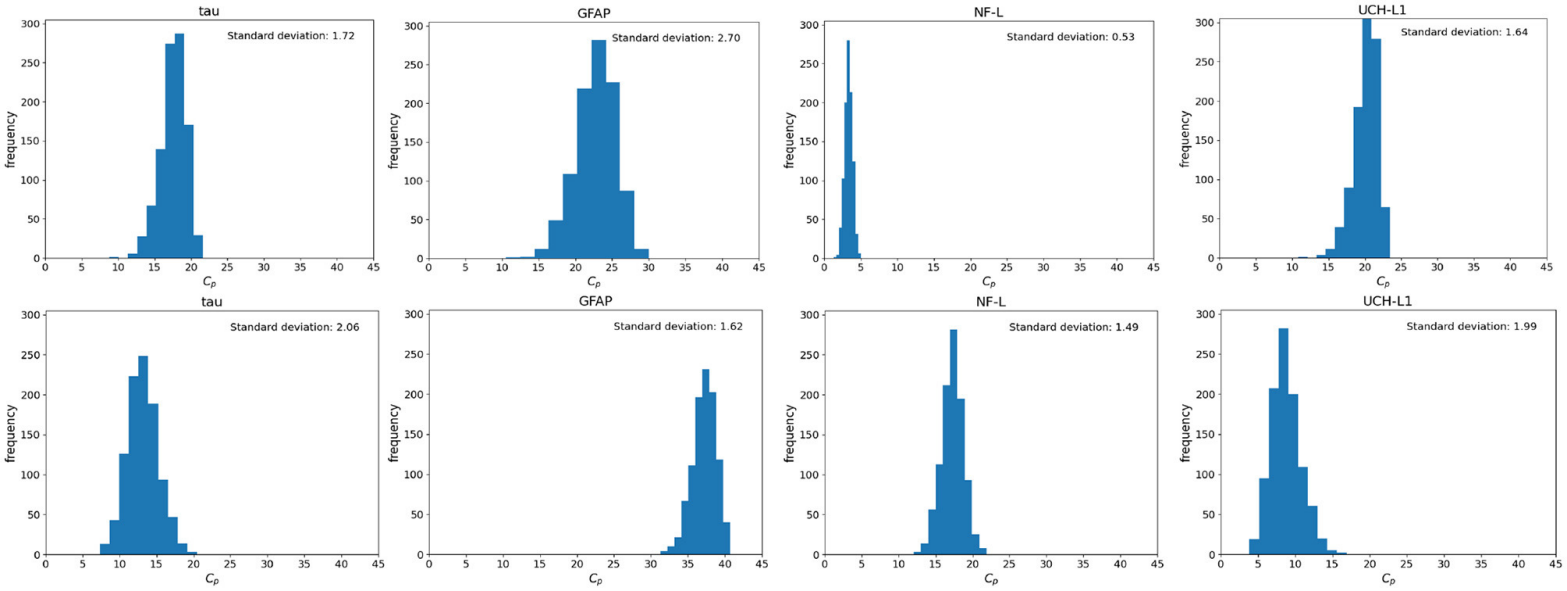
Monte Carlo simulations to investigate the effect of uncertainty in time on the blood biomarker levels (*C*_*p*_). Simulations were conducted by setting the model parameters to the nominal values in Table 1 and the initial biomarker released to *D*_0_ = 400, 000 pg. **(Top)** Histograms of blood biomarker levels at 6 h, with time varying in a range of (3, 12). **(Bottom)** Histograms for blood biomarker levels at 36 h, with time varying in the range of (35, 56). Note that at 36 h, biomarker levels of GFAP and NF-L are still rising while levels of tau and UCH-L1 are falling.

There is often uncertainty as to the exact timing of blood samples after mTBI. Even if the exact timing of blood samples is known it may not be possible to draw the 6 h sample at exactly 6 or 48 h sample at exactly 48 h. Since timing of blood samples can be uncertain or imprecise, we considered two scenarios: a 6-h sample drawn at 6 ± 3 h and a 36 h sample drawn at 36 ± 12 h. Using a Monte Carlo simulation and a nominal *D*_0_ = 400,000 pg we calculated the distribution of *C*_*p*_ at 6 and 36 h (Figure 8). As shown, variations from the designated time of measurement has significant effects on measured biomarker levels, with the exception of the 6-h measurement of NF-L (Figure 8).

### Model Uncertainty Due to Uncertainty About *V*_*d*_

**Figure 9:**
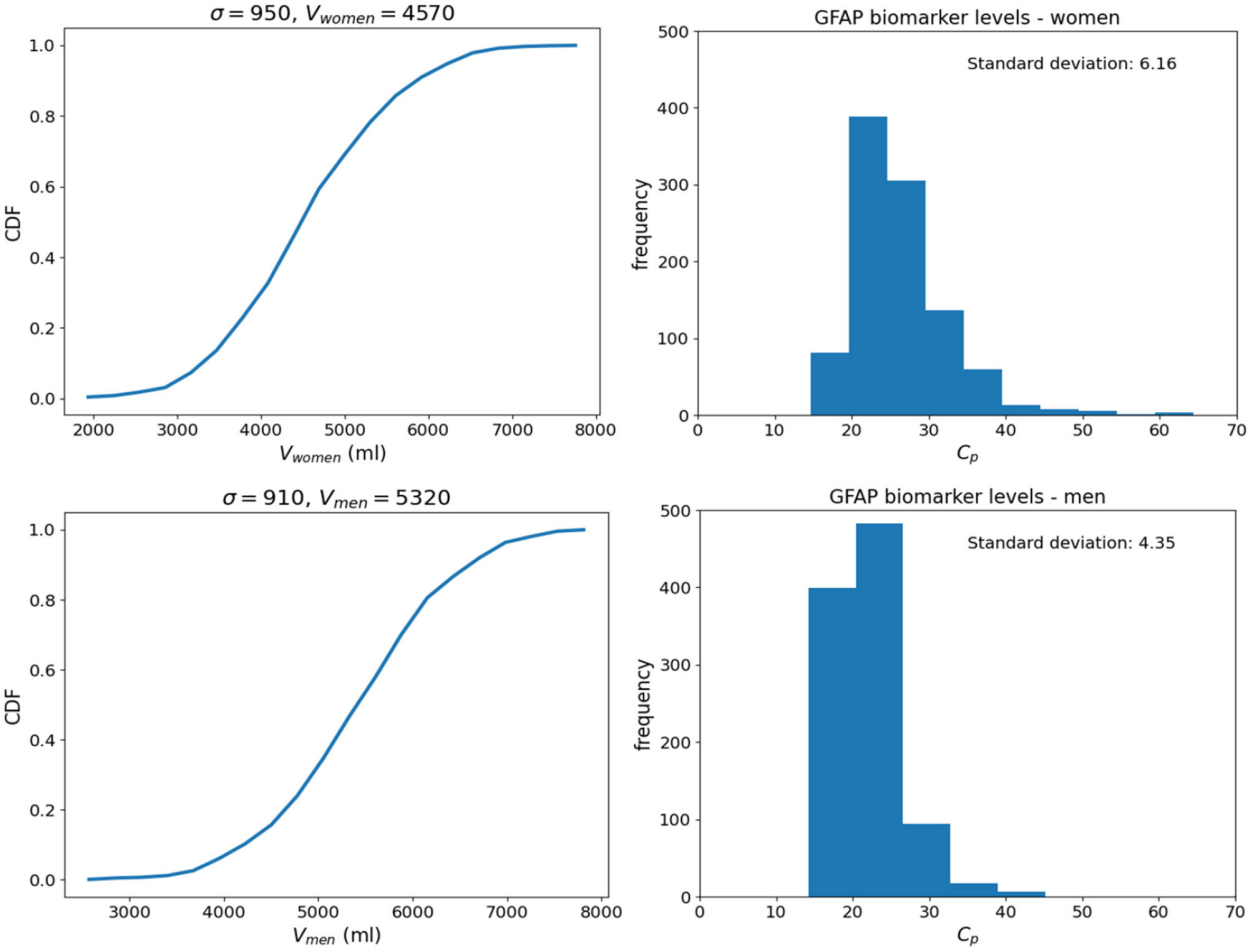
Monte Carlo simulations to investigate the effect of uncertainty in total blood volume on the blood biomarker level (*C*_*p*_). Simulations set the model parameters to the nominal values in Table 1 and a biomarker release of 400, 000 pg. Normal probability distributions with nominal values of 4,750 and 5, 320 ml were used for women and men, respectively. **(Top)** Histograms for GFAP blood biomarker levels at 6 h for women. **(Bottom)** Histograms of tau blood biomarker levels at 6 h for men. Means and standard deviations for blood volumes from published estimates (64).

Since most of the blood biomarkers for mild TBI stay in the blood compartment until renal elimination, it is reasonable to equate the volume of distribution (*V*_*d*_) to the total blood volume. Although total blood volume varies by height, weight, and sex, Feldschuh and Enson (64) corrections for total blood volume are not routinely made. We did 1,000 runs of Monte Carlo simulations on blood levels for the biomarker GFAP at 6 h using the mean total blood volume ± standard deviation for healthy men and women (64) and the nominal kinetic parameters for the tau kinetic model (Table 2). The failure to account for the total blood volume of subjects (Figure 9) introduces considerable variability into modeled biomarker level.

### Kinetic Modeling on Actual Biomarker Data

**Figure 10:**
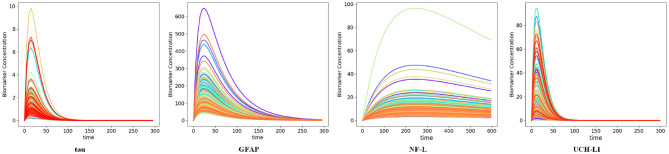
Time-concentration curves for concussed subjects with available biomarker data as estimated by the kinetic models. Each curve represents one subject in the data. Note resemblance of displayed curves to nominal time-concentration curves shown in Figure 3.

**Figure 11:**
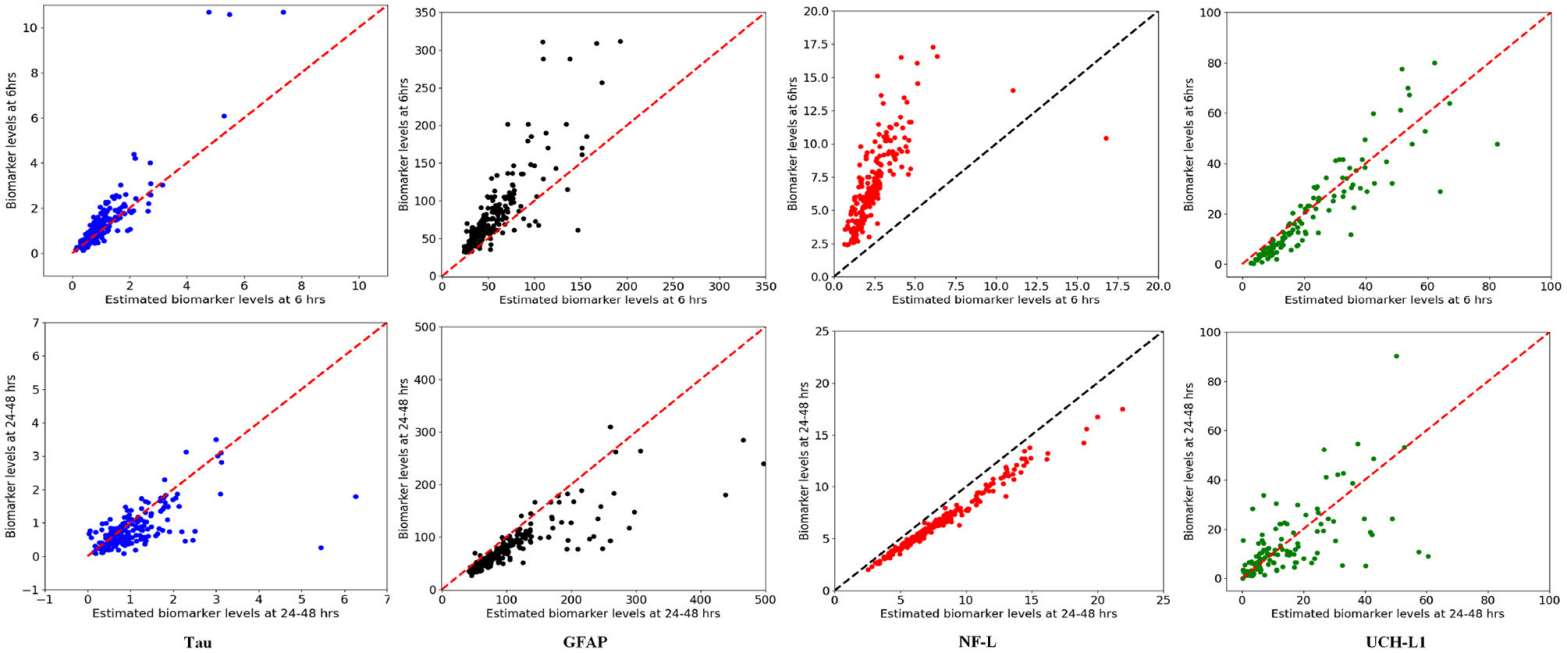
Model estimated biomarker level at 6 and 24–48 h vs. actual biomarker level for each subject using available data set. **(Top)** x-axis corresponds to estimated *C*_*p*_ at 6 h and y-axis corresponds to measured *C*_*p*_ at 6 h. **(Bottom)** x-axis corresponds to estimated *C*_*p*_ at 24–48 h and y-axis corresponds to measured *C*_*p*_ at 24–48 h. Note that estimated levels for NF-L at 6 h are especially below measured levels.

**Figure 12:**
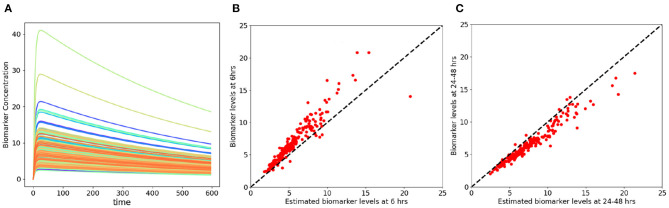
NF-L kinetic modeling utilizing modified kinetic parameters of *T*_*max*_ = 24 h and *k*_*a*_ = 0.2 *hr*^−1^. **(A)** Time concentration curves estimated by the revised kinetic parameters for NF-L biomarker. **(B,C)** Model estimated biomarker level vs. measured biomarker level at 6 and 24–48 h for revised kinetic parameters of NF-L. **(B)** x-axis corresponds to model estimated *C*_*p*_ at 6 h and Y-axis corresponds to measured *C*_*p*_ at 6 h. **(C)** x-axis corresponds to model estimated *C*_*p*_ at 24–48 h and y-axis corresponds to measured *C*_*p*_ at 24–48 h.

We used available biomarker data from the NCAA-CARE study (33) to estimate initial biomarker release (*D*_0_) in the concussed subjects. The data set had values for GFAP, UCH-L1, NF-L, and tau blood biomarkers at four specific time-points for 356 concussed subjects including subjects with more than one concussion. Date stamps included: <6 h post-injury, 24–48 post-injury, when asymptomatic, and 7 days after return to play. Subjects with missing date stamps were excluded yielding a smaller subset for analysis. The total number of mTBI subjects with date stamps available were *n* = 220 for GFAP, *n* = 216 for tau, *n* = 220 for NF-L, and *n* = 123 for UCH-L1. The blood levels available to us were date stamped but not time stamped, so estimates of sampling times were necessarily approximate. We used Equation (3) to estimate *D*_0_ based on *C*_*p*_ at the four available time points. A least-square error method was used to estimate *D*_0_ by minimizing the error between the four blood biomarker measurements and the fitted curve specified in Equation (3). The estimated biomarker released for each subject was substituted into Equation (3) to generate time-concentration curves for all available subjects (Figure 10). Figure 11 shows *measured* blood level (*C*_*p*_) along the y-axis vs. *model estimated* biomarker level (*C*_*p*_) along the x-axis at 6 h (Top row) and 24–48 h (Bottom row). Visual inspection of Figures 10, 11 shows that kinetic profiles follow the general idealized kinetic profile shown in Figure 3. However, a minority of the subjects show higher peak values for *C*_*max*_ compared to the others, suggesting that the distribution of kinetic profiles was bimodal. Additional statistical testing has shown bimodality (submitted for publication). Estimated levels of NF-L, especially at 6 h, fall below measured levels (Figure 11) suggesting an error in model parameters for NF-L. Based on our prior sensitivity analysis (Figure 5) we suspected our approximation of *k*_*a*_ was too low and that actual absorption of NF-L was occurring more rapidly than implied by the model. We re-ran the model for NF-L with a shorter *T*_*max*_ of 24 h and a larger *k*_*a*_ of 0.2 *hr*^−1^, resulting in a better qualitative fit of the estimated *C*_*p*_ to compared to measured *C*_*p*_ (Figure 12).

## Discussion

Biomarkers (biological markers) belong to a broad category of medical signs that are objective indicators of a patient's medical state and that can be measured accurately and reproducibly (70). The intent of obtaining blood biomarkers after mild TBI (concussion) is to address diagnostic questions (Did the subject have a concussion? How severe was the concussion? Should the subject have a CT scan or MRI scan?) or prognostic questions (Can the athlete return to play immediately? When can the withheld athlete return to play? Will the athlete develop post-concussion syndrome?) Ideally the blood biomarker level is a useful surrogate for the brain injury that occurred with concussion. Furthermore, if it could be known, the amount of biomarker released with concussion should be a better measure of severity than a blood biomarker level at a single time-point. A single biomarker level is of limited value. Because blood biomarker levels are changing over time (Figure 3) a blood biomarker level can only be interpreted in the context of its time of measurement. Unlike a blood biomarker level *C*_*p*_ which is variable, the amount of biomarker released at the time of impact is fixed and time constant. If *D*_0_ could be known or estimated, it would be a time-independent correlate to the severity of traumatic brain injury.

Kinetic modeling offers an approach to understanding the complexities and uncertainties in the use of single blood biomarker levels for the diagnosis of mild TBI. For each of five commonly investigated biomarkers for mTBI, we created a one-compartment kinetic model to predict blood biomarker levels at a given time *t* (Figure 3). In general, the kinetic profiles are aligned with suggested kinetic profiles in the published literature (8, 12, 16, 17, 71, 71, 72).

An important limitation of current blood biomarker testing is that blood sampling is frequently not done at standardized times. We used uncertainty analysis to assess the effects of uncertainty about the time of blood sampling (Figure 8). The results suggest that precise timing of blood samples is important to obtaining levels of *C*_*p*_ that reflect *D*_0_ after mild traumatic injury. If blood sampling cannot be done at standardized times, adjustment of measured blood biomarker levels for off-standard times may be helpful, especially when measured biomarker levels are applied to established cut-offs.

Similarly, blood biomarker levels after traumatic brain injury are not currently corrected for individual differences in renal function or total blood volume. Impaired renal function could decrease clearance of blood biomarkers and be reflected in declines in *k*_*e*_ (55). Both the sensitivity analysis for *k*_*e*_ (Figure 5) and the uncertainty analysis for *k*_*e*_ (Figure 7, lower band) suggest that changes in *k*_*e*_ could elevate biomarker levels after mTBI. Although blood biomarker levels are not routinely corrected for individual differences in total blood volume (a surrogate for *V*_*d*_), our sensitivity analysis (Figure 5) and uncertainty analysis (Figure 9) again suggest that variations in total blood volume could significantly change measured levels of biomarker after mTBI. These findings raise the question as to whether corrections in measured *C*_*p*_ after mTBI should be made for renal impairment or blood volume.

A limitation of our kinetic models (Table 2) is the approximate values for *k*_*a*_ and *k*_*e*_. Sensitivity analysis suggests that the model output may be relatively insensitive to errors in our estimates of *k*_*e*_ and *k*_*a*_ except at certain time measurement points that vary by biomarker (Figure 5). Uncertainty analysis (Figure 7) largely confirms the findings of the sensitivity analysis. The model parameters *k*_*a*_ and *k*_*e*_ are important but the model may be relatively resistant to small errors (<30%) in estimates of *k*_*a*_ and *k*_*e*_ (Figure 7). Literature estimates of *T*_*max*_ and half-life for NF-L are especially sparse and unreliable (Table 1). Modeling of levels of NF-L using available data suggested our initial estimates of *T*_*max*_ were too high and our estimate of *k*_*a*_ was too low (Figure 11). Figure 12 shows that revising upward the value of *k*_*a*_ improves model fit with measured available biomarker data.

The kinetic models demonstrate the different concentration-time profiles for each biomarker (Figure 7). These kinetic profiles have implications for selecting the preferred sampling times to obtain the most instructive biomarker levels. In general, it would appear desirable to time blood sampling close to time *T*_*max*_ when *C*_*p*_ is at its maximum *C*_*max*_. Since *T*_*max*_ differs by biomarker (Table 1), preferred sampling time differs by biomarker. Early times should be best for S100B. In the 6–48 h window, UCH-L1 and tau peak earlier than GFAP. Later times seem best for sampling NF-L (Figure 7). These suggestions require empirical verification. More precise kinetic studies of blood biomarker levels after mild TBI (with more frequent and carefully timed and spaced sampling intervals) could provide better estimates of *T*_*max*_, half-life, *k*_*a*_, and *k*_*e*_ and allow more accurate estimations of best sampling times and best cut points.

One important feature of the proposed kinetic models is to work backward from measured levels of biomarker levels *C*_*p*_ to the amount of biomarker released at impact *D*_0_ (Equation 3). Sensitivity analysis by partial correlation (Figure 5) and uncertainty analysis using Monte Carlo simulations (Figures 6B,G) show a strong correlations between measured levels of *C*_*p*_ and the estimation of *D*_0_ at all time intervals. This is a desirable characteristic of the kinetic models and suggests that the models can approximate *D*_0_ at a variety of different time intervals if *t* and *C*_*p*_ are known. We considered the problem of whether a blood biomarker level at a given time *t* could be used to estimate the initial amount of biomarker released at impact (Figure 11). Our thinking is that the amount of biomarker released is a more reliable indicator of brain injury than is a single biomarker level at a single time-point. Although we have demonstrated the feasibility of this approach, further work is needed to explore its utility.

Models can be wrong in at least two ways: their predictions can be wrong or their underlying assumptions can be wrong (73). Our model has several underlying assumptions that could prove wrong. We have assumed that the release of biomarker at impact is momentaneous. In fact, the release of biomarker could occur more slowly over minutes or hours suggesting a delayed release of timed-release model would be more appropriate. Furthermore, studies of biomarker levels after mild traumatic injury have not excluded the possibility of either continuing synthesis of biomarker or upregulation of biomarker synthesis.

We have assumed that blood biomarker levels could be modeled as a one-compartment model (with biomarker entering the blood compartment from the brain and exiting the blood by renal elimination), However, for S100B in particular, the assumptions of the one-compartment model are likely violated as S100B has significant extra-cerebral sources and may be redistributed to the fat and other soft tissues prior to renal elimination (8, 9).

We have assumed that the absorption rate constant *k*_*a*_ is constant over time. Since absorption from the brain likely reflects multiple pathways (perivascular, glymphatic, direct breach of the blood brain barrier, absorption from CSF from blood, etc.), the absorption rate constant *k*_*a*_ could vary over time and likely varies from individual to individual.

Other limitations of this study deserve mention. Our estimate of F, the absorption rate fraction, is especially prone to error since reliable data for estimating F from simultaneous studies of blood and interstitial fluid levels are unavailable to us. Our estimates of the absorption rate constant *k*_*a*_ and the elimination rate constant *k*_*e*_ would have been better if formal kinetic studies of biomarker levels with carefully time sampling were available. A further limitation is that we were not able to recommend specific sampling times and optimal cut points for each blood biomarker. Preferred sampling time depends primarily on knowing the maximum biomarker level *C*_*max*_ at time *T*_*max*_ (or on working backward to *C*_*max*_ from an accurate measure *C*_*p*_ at time t combined with an accurate kinetic model). In the absence of highly accurate kinetic estimates of *k*_*a*_ and *k*_*e*_, we are reluctant to recommend a preferred sampling time for each blood biomarker. However, we suggest that preferred sampling times are likely at or close to their *T*_*max*_. Kinetic modeling could assist in the selection of biomarker cut points for diagnostic testing. However, the cut point chosen will depend upon the proposed use (e.g., discrimination between concussed and non-concussed subjects, identification of concussed subjects with abnormal neuroimaging, identification of subjects with risk of prolonged return to play, etc.).

We have begun to explore the predictive power of a kinetic model by comparing actual and predicted blood biomarker levels in an actual available data set (Figure 11). Although the kinetic model fits the general pattern of blood biomarker levels after mTBI based on the real data (Figure 10), the lack of exact time-points for blood biomarker measurements has limited our ability to do an exact goodness of fit determination. At present we conclude that our model fits the NCAA-CARE dataset qualitatively (Figure 11). We have already commented above that our initial approximation for *k*_*a*_ for NF-L was likely too low (Figure 12). Modification of model parameters could improve goodness of fit (Figure 12).

Like McCrea et al. (33), we noted that the biomarker levels after mild TBI in the NCAA-CARE dataset were heteroscedastic and likely not normal. We elected not to log transform the data to persevere its bi-modality which potentially reflected two distinct populations after mild TBI. Further investigation of the heteroscedasticity of blood biomarker levels after mild TBI is needed but is beyond the scope of this paper.

Several improvements in the proposed model are possible. One improvement would be to add in corrections for subject age, height, weight, and renal function. Our current model is based on increases in biomarker levels due to mTBI and does not consider the baseline levels of these biomarkers in control subjects. The model could be improved by adding back in corrections for baseline levels of biomarkers.

In conclusion, we have demonstrated the feasibility of creating a one-compartment kinetic model of blood biomarker levels after mild traumatic brain injury. The one-compartment kinetic model fits well the observed levels of tau, UCH-L1, and GFAP after mild traumatic brain injury. The accuracy of the key kinetic parameters of the kinetic model (*k*_*a*_, *k*_*e*_, and *F*) could be improved by carefully executed kinetic studies. S100B and NF-L pose special challenges to modeling. S100B is likely absorbed into the blood from multiple compartments and may be redistributed to other compartments prior to elimination. Hence, S100B may be a poor candidate for a one-compartment kinetic model. The paucity of kinetic data on NF-L added to the possibility of late release or NF-L or the upregulation of NF-L synthesis, suggests that a different model may be needed for NF-L biomarker levels. Additional careful kinetic studies of biomarker levels could resolve the issue as to whether release at impact is momentaneous or stage-released. Accurate kinetic models of blood biomarker levels have the potential to improve the selection of optimal sampling times and optimal cut points for the blood biomarkers used in the diagnosis of mild TBI.

## Data Availability Statement

Publicly available datasets were analyzed in this study. The CARE biomarker data utilized in this study are available to approved investigators via the FITBIR (32) data repository, https://fitbir.nih.gov/. The data utilized for the kinetic model design are available upon request to the corresponding author.

## Author Contributions

SA, DH, BA, TO-A, GO, MT, and DW: concept and design, data interpretation, drafting, revising, and final approval. TO-A: data acquisition. SA and DH: model parameters and computations. All authors contributed to the article and approved the submitted version.

## Conflict of Interest

The authors declare that the research was conducted in the absence of any commercial or financial relationships that could be construed as a potential conflict of interest.
